# SIRT1 activating compounds reduce oxidative stress and prevent cell death in neuronal cells

**DOI:** 10.3389/fncel.2012.00063

**Published:** 2012-12-31

**Authors:** Reas S. Khan, Zoe Fonseca-Kelly, Catherine Callinan, Ling Zuo, Mira M. Sachdeva, Kenneth S. Shindler

**Affiliations:** ^1^Scheie Eye Institute and F.M. Kirby Center for Molecular Ophthalmology, University of PennsylvaniaPhiladelphia, PA, USA; ^2^Department of Ophthalmology, School of Engineering, University of PennsylvaniaPhiladelphia, PA, USA; ^3^Department of Ophthalmology, Second Hospital of Jilin UniversityJilin, China; ^4^Department of Ophthalmology, Wilmer Eye Institute, Johns Hopkins HospitalBaltimore, MD, USA

**Keywords:** resveratrol, SIRT1, neuroprotection, oxidative stress, optic neuropathy, mitochondria

## Abstract

Activation of SIRT1, an NAD+-dependent deacetylase, prevents retinal ganglion cell (RGC) loss in optic neuritis, an inflammatory demyelinating optic nerve disease. While SIRT1 deacetylates numerous protein targets, downstream mechanisms of SIRT1 activation mediating this neuroprotective effect are unknown. SIRT1 increases mitochondrial function and reduces oxidative stress in muscle and other cells, and oxidative stress occurs in neuronal degeneration. We examined whether SIRT1 activators reduce oxidative stress and promote mitochondrial function in neuronal cells. Oxidative stress, marked by reactive oxygen species (ROS) accumulation, was induced in RGC-5 cells by serum deprivation, or addition of doxorubicin or hydrogen peroxide, and resulted in significant cell loss. SIRT1 activators resveratrol (RSV) and SRTAW04 reduced ROS levels and promoted cell survival in RGC-5 cells as well as primary RGC cultures. Effects were blocked by SIRT1 siRNA. SIRT1 activators also increased expression of succinate dehydrogenase (SDH), a mitochondrial enzyme, and promoted deacetylation of PGC-1α, a co-enzyme involved in mitochondrial function. Results show SIRT1 activators prevent cell loss by reducing oxidative stress and promoting mitochondrial function in a neuronal cell line. Results suggest SIRT1 activators can mediate neuroprotective effects during optic neuritis by these mechanisms, and they have the potential to preserve neurons in other neurodegenerative diseases that involve oxidative stress.

## Introduction

Optic neuritis is an inflammatory demyelinating optic nerve condition that often occurs in multiple sclerosis (MS) patients (Arnold, 2005). Axonal loss also occurs in optic neuritis patients and correlates with decreased vision (Trip et al., 2005; Fisher et al., 2006). Significant loss of retinal ganglion cells (RGCs), the neurons that comprise the optic nerve, occurs in experimental models of optic neuritis (Guan et al., 2006; Shindler et al., 2006; Quinn et al., 2011), providing useful models of neuronal degeneration in demyelinating disease of the central nervous system. Recent studies demonstrated that RGC and optic nerve injury are accompanied by increased reactive oxygen species (ROS) (Lieven et al., 2006), predominantly in mitochondria, the primary source of cellular ROS production and detoxification (Andreyev et al., 2005). Increased ROS is linked to neurodegenerative diseases (Kalman and Leist, 2003), including optic neuropathies (Qi et al., 2003, 2007). Although ROS are key mediators of CNS injury in MS and its animal models (Cowden et al., 1998; Mohamed et al., 2003), contributions of mitochondria to ROS activity and cell death are not well understood.

SIRT1 is a member of the sirtuin gene family encoding NAD+-dependent deacetylases that deacetylate histones and prolong survival (Imai et al., 2000; Baur et al., 2006). Sirtuins deacetylate numerous protein targets involved in various cellular pathways, including stress responses, apoptosis, and axonal degeneration (Yang and Sauve, 2006). Sirtuin activators and inhibitors modulate SIRT1 activity by altering the K_m_ for its substrates (Baur, 2010). We previously showed three distinct SIRT1 activators attenuate RGC loss induced by experimental optic neuritis (Shindler et al., 2007, 2010; Fonseca-Kelly et al., 2012). The mechanism of this neuroprotection is not fully understood, but is dependent on SIRT1, as SIRT1 inhibitors block the protective effects, and the mechanism does not involve suppression of inflammation, suggesting other pathways must be involved.

Resveratrol (RSV) is a phytoalexin present in numerous plants (Dong, 2003). RSV has multiple protective effects including cardioprotection (Chu et al., 2011), antiaging (De la Lastra and Villegas, 2005), and defense against metabolic and neurodegenerative diseases (Pallàs et al., 2009). RSV improves mitochondrial function and protects against metabolic disease by activating SIRT1 to deacetylate the mitochondrial co-enzyme PGC-1α (Lagouge et al., 2006). In addition, RSV exhibits antioxidant activities (Sun and Spranger, 2005), ROS scavenging (Burkitt and Duncan, 2000), and regulates antioxidase expression (Murias et al., 2005). RSV affects different metabolic pathways depending on cell type, cell state, and duration and dosage of treatment. At the same time, RSV has numerous intracellular targets including transcription factors/cofactors, thus regulating metabolic homeostasis and resulting in multiple pleiotropic effects (Barger et al., 2008; Wang et al., 2009).

Therefore, it is important to determine specific signaling pathways that RSV and other SIRT1 activators promote in neurons, to better understand mechanisms of their neuroprotective effects. Unfortunately, no specific RGC cell line exists. In the present study, the ability of SIRT1 activators to prevent cell death induced by oxidative stress was evaluated using RGC-5 cells. While the RGC-5 cell line was originally reported to be a transformed rat cell line with features of RGCs (Krishnamoorthy et al., 2001), it has since been recharacterized (Van Bergen et al., 2009), demonstrating features of mouse photoreceptors. RGC-5 cells retain some characteristics of neurons, including differentiation into neuronal-like cells with neurite outgrowth induced by treatment with staurosporine (Frassetto et al., 2006; Wood et al., 2010). The ability of two SIRT1 activators, RSV and SRTAW04, to promote SIRT1 activity, reduce ROS accumulation, and increase markers of mitochondrial function was assessed in RGC-5 cells, and because of the limits of the RGC-5 cell line, key effects were also examined in primary RGCs isolated from neonatal mice.

## Materials and methods

### Mice

Six-week-old female SJL/J mice were purchased from the Jackson Laboratory (Bar Harbor, ME) for induction of experimental optic neuritis. For isolation of primary retinal cells, 3–5-day-old C57/Bl6 mouse pups were removed from litters of timed-pregnant mice (Jackson). Treatment of animals was reviewed and approved by the Institutional Animal Care and Use Committee at the University of Pennsylvania.

### Experimental autoimmune encephalomyelitis (EAE)

EAE was induced as previously described (Shindler et al., 2006). Briefly, eight-week-old female SJL/J mice were anesthetized with ketamine/xylazine and were injected subcutaneously at two sites on the back with 0.1 mL solution containing 0.5 mg/mL proteolipid protein peptide 139–151 (GenScript, Piscataway, NJ) emulsified in complete Freund adjuvant (CFA; Difco, Detroit, MI) containing 2.5 mg/mL Mycobacterium tuberculosis (Difco). Control mice were injected with equal volumes of PBS and CFA. All mice were injected with 200 ng pertussis toxin (List Biological, Campbell, CA) in 0.1 mL PBS intraperitoneally on day 0 (day of immunization) and again on day 2.

### RGC-5 culture and viability

Cultures of RGC-5 cells (ATCC, Manassas, VA) were maintained in growth medium containing low-glucose Dulbecco's modified Eagle's medium (DMEM) containing 10% fetal bovine serum, 100 U/mL penicillin, and 100 μg/mL streptomycin (Sigma-Aldrich, St. Louis, MO) in a humidified atmosphere of 95% air and 5% CO_2_ at 37°C, as described by Krishnamoorthy et al. (2001). Cells were passaged every 3–4 days, with a doubling time of 18–20 h. During each experiment, cells were plated at a concentration of 1 × 105 cells/mL and after 16 h treated with various concentrations of RSV (Sigma-Aldrich) or SRTAW04 (Sirtris, a GSK company, Cambridge, MA) dissolved in DMSO with or without H_2_O_2_ for 24 h. To obtain staurosporine-differentiated RGC-5 cells, cells were exposed to 1 μM staurosporine (Cayman Chemical, Ann Arbor, MI) for 6 h, washed three times with PBS and then recovered in culture medium for 1 day. Control cells were treated with DMSO only. DMSO concentration in each well was below 0.5% v/v. For doxorubicin experiments, cells were plated at 1 × 105 cells/mL and after 16 h treated with 1 μM of doxorubicin (Sigma, St. Louis, MO) for another 24 h. For serum starvation experiments, media were replaced with serum free media and cells were grown for 24 h. Cell viability was measured by trypan blue staining and counted on a hemocytometer. Alternatively, cell viability was assessed using PrestoBlue™ Cell Viability Reagent (Invitrogen, Grand Island, NY). Twenty-two microliter was added to each well and incubated for 20 min, and fluorescence was read on a plate reader excited at 570 nm with non-conjugated light emissions collected at 610 nm.

### Primary RGCs

The retina was removed from 3- to 5-day old C57/Bl6 mice and cells were dissociated as described previously for neonatal rat retina (Heng et al., 1999). Briefly, retinas were dissociated in solution containing 0.45 U papain (Worthington, Lakewood, NJ) for 30 min at 37°C. After digestion, the retinal tissue was rinsed twice with DMEM before being transferred into Neurobasal medium containing 1% BSA (Sigma) B27-supplement (1:50, Gibco) and penicillin/streptomycin (Gibco). Then, tissue was triturated and passed through a cell strainer before seeding of the isolated cells onto poly-D-lysine (0.1 mg/ml, molecular weight <300,000 Da, Sigma) and laminin (20 μg/ml, Sigma) coated chamber slides. To quantify RGC survival, chamber slides were washed twice with PBS and fixed in 4% paraformaldehyde for 15 min. Fixed cells were permeabilized using 0.3% Triton X-100 and blocked with 1% BSA in PBS for 1 h followed by incubation with anti Brn3a antibody (Santa Cruz biotechnology, Santa Cruz, CA) over night at 4°C. After washing with PBS, fluorescence-labeled secondary antibodies (Invitrogen) were applied for 1 h at room temperature (RT). Finally, cells were embedded in mounting medium containing DAPI (Vector labs, Burlingame, CA), pictures were taken using a fluorescence microscope (Eclipse E600; Nikon, Tokyo, Japan), at 10× magnification and the Brn3a specific RGCs were counted by a masked investigator.

### MitoSOX staining

MitoSOX Red (Invitrogen) mitochondrial superoxide indicator is a fluorogenic dye for selective detection of superoxide in the mitochondria of live cells. For RGC-5 cultures, MitoSOX reagent was diluted to a final concentration of 3 μM in warm DMEM 1% FBS and added to the cells. After 15 min incubation at 37°C, cells were fixed with 4% paraformaldehyde for 10 min, mounted onto glass slides with Mowiol mounting medium, and observed under a Eclipse E600 (Nikon, Tokyo, Japan) fluorescence microscope using excitation 510 nm/emission 580 nm. For optic nerves, mice were anesthetized with ketamine/xylazine and the optic nerves were removed, washed with PBS and incubated in 5 μM MitoSOX Red for 30 min at 37°C. After incubation, nerves were washed three times with PBS and mounted in OCT. Five micromole cross-sections were made, viewed by fluorescent microscopy, and photographed at 20× magnification. One photograph centered within each optic nerve cross section was taken by a blinded investigator using a standard exposure, and staining was quantified by calculating the optical density using Image J software (nih.gov).

### Western blot

Cell cultures were washed twice with PBS, trypsinized for 5 min, diluted with 10× volume of PBS and centrifuged at 2000g for 10 min. Pellets were lysed in RIPA buffer (150 mM NaCl, 1% NP-40, 0.5% desoxycholic acid, 0.1% SDS and 50 mM Tris, pH 8) to obtain total protein, and protein content was measured with the BCA protein assay kit (Thermo scientific, Rockford, IL). Proteins (30 μg) were separated by polyacrylamide gel electrophoresis using 10% SDS–polyacrylamide gels under reducing conditions, then transferred with semidry blotting (Bio-Rad, Hercules, CA) to Nitrocellulose High bound ECL membranes (GE Healthcare Biosciences, Pittsburgh, PA). Membranes were blocked with Odyssey Blocking Buffer (Licor Biotechnology, Lincoln, NE) for 1 h at RT. Rabbit polyclonal antibodies against SIRT1 (AbCam, Cambridge, MA) were used at a 1:1500 dilution overnight at 4°C. As secondary antibody, IRDye® 800CW Goat anti-rabbit IgG (Licor) was incubated at a 1:5000 dilution for 1 h at RT. Rabbit polyclonal SOD2 (GeneTex, Irvine, CA) and mouse monoclonal succinate dehydrogenase b (SDHb) (Abcam, Cambridge, MA) antibodies were used at 1:1000 dilutions. As secondary antibody, IRDye® 800CW goat anti-rabbit IgG and IRDye® 600 goat anti-mouse IgG were used at a dilution of 1:5000. Fluorescence was visualized using Odyssey infrared imaging system (Licor). For normalization of signals, blotted membranes were stained for β-actin (Sigma). The intensity of each band was determined using Image J software (nih.gov).

### Detection of PGC-1α acetylation

PGC-1α was immunoprecipitated from cell extract (500 μg of nuclear protein) using anti-PGC-1α antibody (Santa Cruz). Immunoprecipitated PGC-1α was electrophoresed in SDS-PAGE and immunoblotted with antibody specific for acetylated lysine (Cell Signaling Technology, Boston, MA) to detect acetylation, or antibody specific for PGC-1α (Novus Biologicals, Littleton, CO) to detect total PGC-1α levels.

### JC-1 staining of mitochondrial membrane potential

JC-1 (Immunochemistry Technologies, Bloomington, MN) staining was performed according to manufacturer's instructions to assess mitochondrial membrane potential (Cossarizza et al., 1993). In healthy cells due to higher mitochondrial membrane potentials, JC-1 forms fluorescent “J aggregates” that appear red. However, JC-1 exists as a monomer at low mitochondrial membrane potential, as seen in apoptotic cells, and appears green. Thus, the emission of this cyanin dye can be used as a measure of mitochondrial membrane potential. A stock solution of JC-1 was prepared at 4 mg/mL in dimethylsulfoxide (DMSO). The stock JC-1 solution was added drop-wise, while vortexing, to control medium to a final concentration of 10 μg/mL. The diluted JC-1 solution was then passed through a 0.2-μM syringe filter (GeneMate, Kaysville, UT) and added to RGC-5 cultures for 15 min at 37°C. After incubation, staining solutions were decanted, each dish was washed three times with Ringer's buffer [in mM; 130 NaCl, 5 KCl, 2 CaCl_2_.2H_2_O, 1 MgSO_4_, 8 NaOH, 1 NaPO_4_, 5.5 D-glucose (pH 7.4)] at 37°C, and cells were left in fresh Ringer's buffer. Live cell images were acquired using the Argon laser on a fluorescent microscope (Eclipse E600; Nikon). JC-1 was excited at 488 nm and non-conjugated light emissions were collected at 530 nm (green) and conjugated at 590 nm (red). The level of JC-1 staining was quantified by photographing cultures with a standard exposure and calculating the optical density of staining using Image J software.

### TMRM staining of mitochondrial membrane potential

TMRM (Immunochemistry Technologies, Bloomington, MN) staining was performed according to manufacturer's instructions. Differentiated RGC-5 cells after treatment were incubated with 100 nM of TMRM for 15 min and washed twice with washing buffer. Live cell images were acquired using the fluorescent microscope (Eclipse E600; Nikon). The level of TMRM staining was quantified by photographing cultures with a standard exposure and calculating the optical density of staining using Image J software.

### SIRT1 inhibition with siRNA

SIRT1-specific siRNA, and control siRNA were Silencer select predesigned siRNAs from Life Technologies (Invitrogen) and experiments were performed according to manufacturer's instructions. Briefly, mouse primary retinal cells or differentiated RGC-5 cells were treated with 25 nm of either SIRT1 specific siRNA or control siRNA using lipofectamine RNA iMAX transfection reagent. 24 h later, the cells were treated with H_2_O_2_ with or without drug treatment and cell survival was assessed 48 h after transfection.

### Statistics

Data are expressed as means ± SEM. For cell viability, SIRT1 activity, and western blot analysis, differences between culture groups were assessed using One-Way ANOVA followed by Student Neuman–Keuls *post-hoc* test. Statistical differences were considered significant at *P* < 0.05.

## Results

### ROS accumulate in optic neuritis and induce toxicity in RGC-5 cells

**Figure 1:**
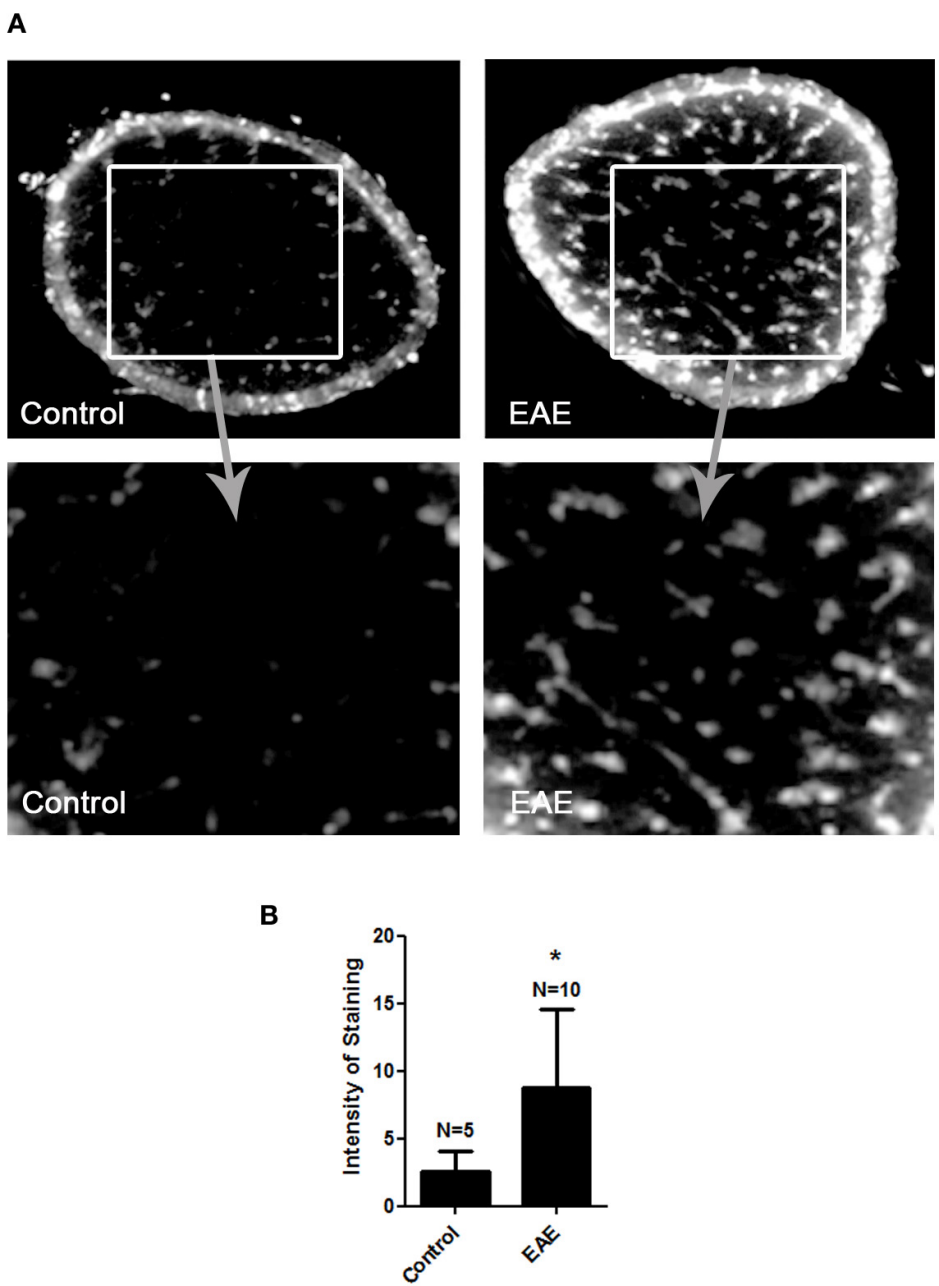
**ROS accumulate in the optic nerve during EAE.** Eight-week-old female SJL mice were immunized with proteolipid protein and were sacrificed 11 days later. Optic nerves of EAE and control mice were isolated and stained with MitoSOX Red. **(A)** Cross-sections show high levels of MitoSOX Red staining, a marker of superoxide anion, throughout the parenchyma of EAE optic nerves, with significantly less staining in control optic nerves. One representative nerve from a control mouse, and one EAE optic nerve, is shown at 10× original magnification (top) and 40× original magnification (bottom). **(B)** The average intensity of MitoSOX staining is significantly higher in EAE optic nerves as compared to control optic nerves (^*^p < 0.05).

MitoSOX Red detection of superoxide within mitochondria was used to confirm prior studies suggesting a role of ROS accumulation in optic neuritis (Qi et al., 2007) in mice with EAE, a model of MS. EAE was induced in female SJL/J mice by immunization with proteolipid protein peptide, and mice were sacrificed 11 days later, when optic nerve inflammation is known to peak (Shindler et al., 2006, 2008). Ten optic nerves of 5 EAE mice and 5 control mouse optic nerves were isolated and incubated with MitoSOX Red. Fluorescent microscopy of cryosectioned EAE specimens revealed an increase in the superoxide anion compared to control optic nerves (Figure 1).

**Figure 2:**
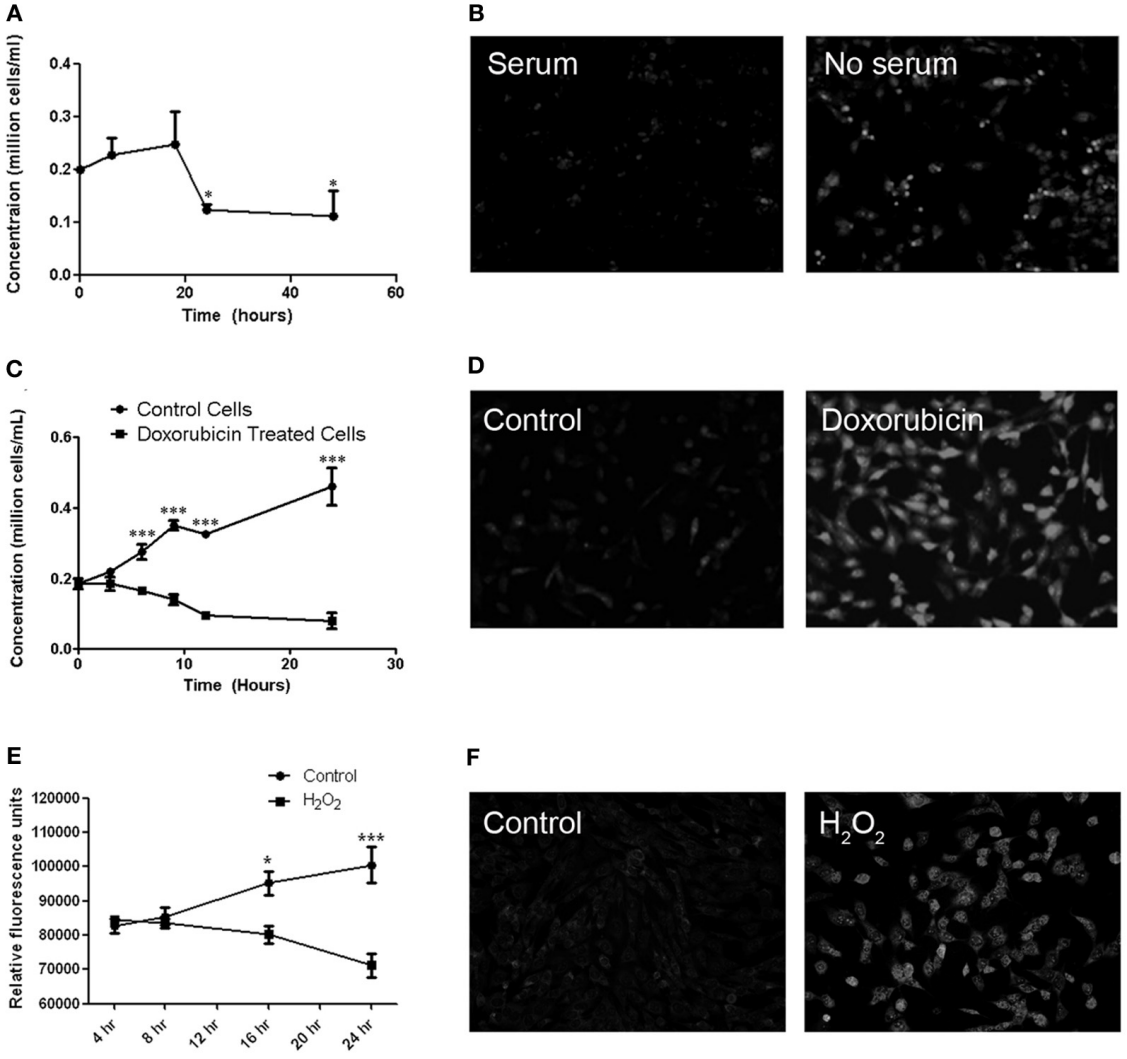
**Cell viability and MitoSOX staining in cultured RGC-5 cells in response to stressors. (A)** RGC-5 cells were plated in serum-containing medium for 16 h and then stressed by serum starvation of RGC-5 cells for the next 48 h. A significant decrease in numbers of viable cells, counted by trypan blue exclusion, (^*^p < 0.05) occurs by 24 h after serum removal. **(B)** There is an associated increase in the number of MitoSOX Red staining cells in serum-deprived cultures as compared to serum-containing cultures. **(C)** RGC-5 cells were plated in serum-containing medium for 16 h and then treated with 1 μM doxorubicin for 24 h. Cell viability was assessed by trypan blue exclusion. Doxorubicin induces a significant decrease (^***^p < 0.001) in RGC-5 cell number compared to control cultures, beginning within 6 h of incubation. **(D)** Increased superoxide staining is observed in doxorubicin treated RGC-5 cultures. **(E)** RGC-5 cells were plated in serum-containing medium for 16 h, then treated with 500 μM H_2_O_2_ for 24 h. Cell viability was assessed using PrestoBlue™ Cell Viability Reagent. A significant decrease in cell viability (^*^p < 0.05; ^***^p < 0.001) occurs after H_2_O_2_ treatment. **(F)** H_2_O_2_ induces an increase in MitoSOX staining.

MitoSOX staining was used to determine whether cultured RGC-5 cells demonstrate similar superoxide accumulation in mitochondria in response to various stressors, as seen in RGCs axons in EAE optic neuritis. Cell viability was also measured. RGC-5 cells were plated and incubated for 16 h prior to being stressed by removal of serum, or by addition of doxorubicin or hydrogen peroxide. Serum starvation of the RGC-5 cells showed a significant decrease in cell viability by 16 h after serum removal, with an associated increase in MitoSOX Red staining (Figures 2A,B). Treatment with 1 μM doxorubicin induced a significant decrease in RGC-5 cells compared to control cultures, beginning within 6 h of incubation, also with a robust increase in the superoxide staining (Figures 2C,D), and similar RGC-5 cell loss and ROS accumulation occurred in cultures treated with 500 μM hydrogen peroxide (Figures 2E,F).

**Figure 3:**
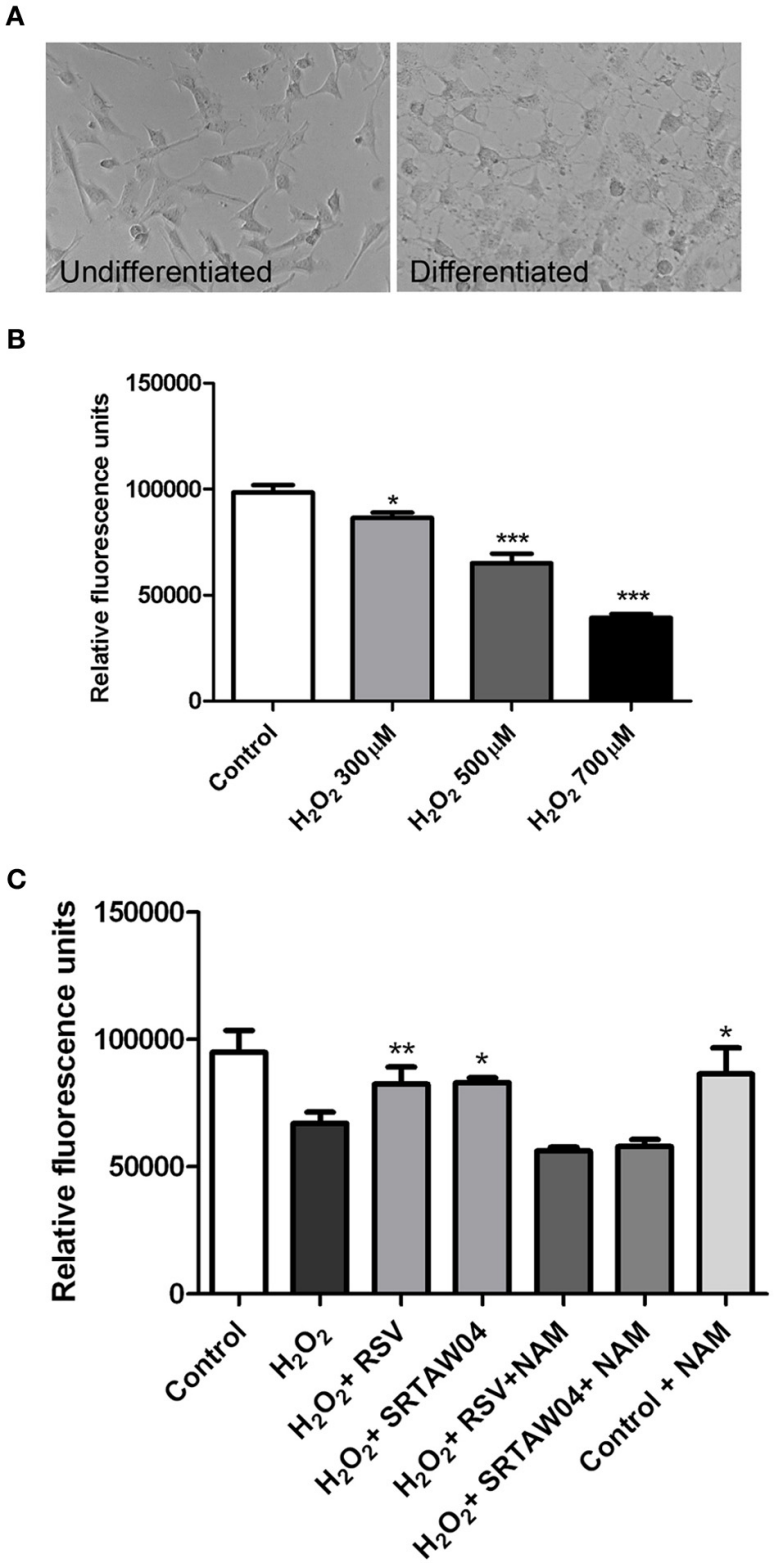
**H_2_O_2_-induced loss of neuronal differentiated RGC-5 cells attenuated by SIRT1 activators. (A)** RGC-5 cells were plated in serum-containing medium for 16 h and then differentiated into neurite-sprouting neuronal cells by addition of 1 μM staurosporine for 6 h. Phase-contrast photographs demonstrate morphologic changes, including presence of neurites, in differentiated cells. **(B)** Twenty-four hours treatment of differentiated RGC-5 cells with varying doses of H_2_O_2_ shows a significant dose dependent decrease in cell viability (^*^p < 0.05 for 300 μM H_2_O_2_ vs. control; ^***^p < 0.001 for 500 μM H_2_O_2_ vs. control; ^***^p < 0.001 for 700 μM H_2_O_2_ vs. control) as measured by PrestoBlue Cell Viability Reagent. **(C)** Pretreatment with 0.25 μM RSV or 5 μM of SRTAW04 beginning 30 min before H_2_O_2_ treatment, and continuing through 24 h exposure to 500 μM H_2_O_2_, shows a significant attenuation of RGC-5 death as compared to cells exposed to H_2_O_2_ alone (^*^p < 0.05; ^**^p < 0.01). The ability of RSV and SRTAW04 to attenuate RGC-5 cell death is blocked by addition of SIRT1 inhibitor NAM (200 μM). NAM alone does not alter cell viability.

Because RGC-5 cells are a transformed, dividing cell line, we next pretreated RGC-5 cells with staurosporine (1 μM for 6 h), which drives neuronal differentiation with sprouting of neurites (Frassetto et al., 2006) (Figure 3A). Serum withdrawal and doxorubicin induce cell loss and ROS accumulation in differentiated RGC-5 cells (data not shown) as in undifferentiated cells (Figure 2). We also directly introduced oxidative stress by treating with hydrogen peroxide and found a dose dependent loss of differentiated RGC-5 cells (Figure 3B). We used hydrogen peroxide (500 μM) for subsequent experiments to directly drive oxidative stress.

### SRTAW04 and RSV attenuate neuronal cell death

Previous studies showed SIRT1 activators significantly attenuate RGC loss in experimental optic neuritis (Shindler et al., 2007, 2010; Fonseca-Kelly et al., 2012), although molecular mechanisms of effects on neuronal survival are not well understood. We investigated whether similar effects can be reproduced in neuronal cultures. RGC-5 cells were differentiated by 1 μM staurosporine for 6 h and then grown in media with 10% serum for 24 h. After 24 h, cells were observed for their morphological features. Their somas became rounder and neurites gradually increased, similar to prior studies (Frassetto et al., 2006). The cells contacted each other with multiple long neurites, features of neuronal differentiation (Figure 3A). Differentiated cells were then pretreated with SIRT1 activating compounds, 0.25 μM RSV or 5 μM SRTAW04, for 30 min before addition of 500 μM H_2_O_2_. Treatment with both SRTAW04 and RSV showed significant reduction of RGC-5 cell death compared to untreated cells, 24 h after H_2_O_2_ addition; treatment with a known SIRT1 inhibitor (Baur, 2010), nicotinamide (NAM; 200 μM), attenuated this protective effect whereas NAM treatment alone did not alter cell viability (Figure 3C). We also evaluated effects of higher RSV and SRTAW04 concentrations on cell survival, but found no increased benefit of RSV at concentrations higher than 0.25 μM, nor SRTAW04 higher than 5 μM (data not shown).

### SRTAW04 and RSV reduce ROS in RGC-5 cells

**Figure 4:**
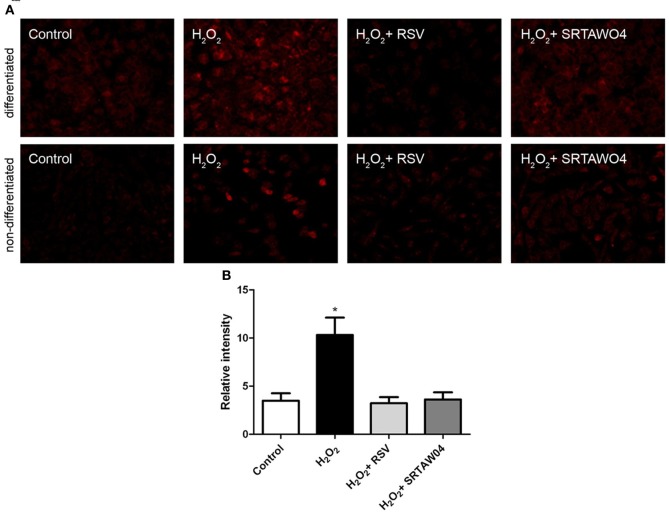
**SRTAW04 and RSV attenuate ROS in RGC-5 cells.** RGC-5 cells were grown for 24 h with or without (control) 500 μM H_2_O_2_, and treated continuously with 0.25 μM RSV or 5 μM SRTAW04 where indicated, beginning 30 min before addition of H_2_O_2_. Cells were stained with MitoSOX Red to assess ROS accumulation. **(A)** Significant attenuation of MitoSOX staining is shown in representative cultures treated with RSV and SRTAW04 compared to untreated H_2_O_2_-containing cultures for both non-differentiated (top) and differentiated (bottom) RGC-5 cells. **(B)** The average intensity of MitoSOX staining is significantly higher in H_2_O_2_ cultures as compared to control cultures (^*^p < 0.05), and compared to RSV (^*^p < 0.05) and SRTAW04 (^*^p < 0.05) treated cultures of differentiated RGC-5 cells.

To investigate whether RSV and SRTAW04 attenuate superoxide accumulation produced by hydrogen peroxide treatment, RGC-5 cells were pretreated with 0.25 μM RSV or 5 μM SRTAW04, as used for the cell viability experiments. Cells were stained with MitoSOX Red 24 h after exposure to 500 μM H_2_O_2_, and experiments were repeated using both differentiated and non-differentiated RGC-5 cells. MitoSOX staining in differentiated cells showed that H_2_O_2_ treatment induces an increase in MitoSOX staining which is attenuated by both RSV and SRTAW04, and non-differentiated RGC-5 cells showed the same effect (Figure 4A). Quantification of the intensity of MitoSOX staining in differentiated RGC-5 cells shows that this effect is significant (Figure 4B). Untreated differentiated cells showed a small increase in MitoSOX Red staining compared to untreated non-differentiated cells, suggesting some effect from staurosporine treatment itself.

### SRTAW04 and RSV attenuate H_2_O_2_-induced loss of mitochondrial membrane potential

**Figure 5:**
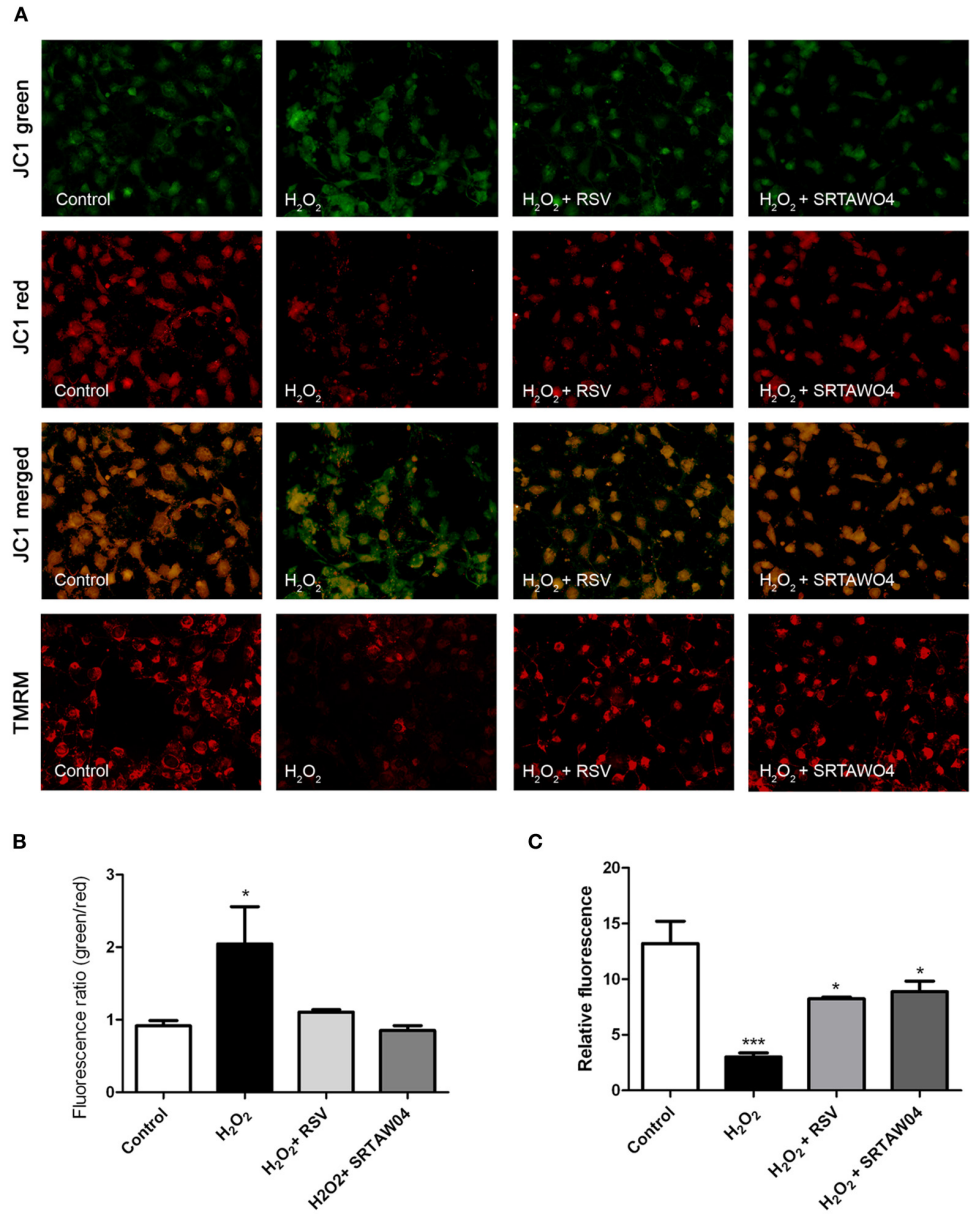
**SRTAW04 and RSV attenuate H_2_O_2_ induced loss of mitochondrial potential.** RGC-5 cells were plated in serum-containing medium for 16 h and then differentiated into neuronal cells by addition of 1 μM staurosporine for 6 h. Differentiated RGC-5 cells were maintained for 24 h with or without (control) 500 μM H_2_O_2_, and treated with 0.25 μM RSV or 5 μM SRTAW04 where indicated, beginning 30 min before addition of H_2_O_2_. JC-1 and TMRM stains were used to assess mitochondrial membrane potential. **(A)** For comparison, a representative field containing similar cell numbers was photographed from each culture (despite overall lower cell numbers in cultures containing H_2_O_2_ without SIRT1 activators). Green JC-1 staining (top row) labeling all mitochondria, including those with low membrane potential, demonstrates similar mitochondrial numbers in each culture. Red JC-1 staining (second row) labeling of normal mitochondria shows significant loss of membrane potential in cells exposed to H_2_O_2_, and this is reversed by addition of RSV or SRTAW04. Merged images of green and red JC-1 staining (third row) show a high green to red ratio in cells exposed to H_2_O_2_, with a lower ratio following addition of RSV or SRTAW04. Red TMRM staining (bottom row) of normal mitochondria shows similar loss of membrane potential in cells exposed to H_2_O_2_ that is reversed by addition of RSV or SRTAW04. **(B)** The average ratio of green to red staining of JC-1 is significantly higher in H_2_O_2_ cultures as compared to control cultures (^*^p < 0.05), and compared to RSV (^*^p < 0.05) and SRTAW04 (^*^p < 0.05) treated cultures. **(C)** The average intensity of TMRM staining is significantly lower in H_2_O_2_ cultures as compared to control cultures (^***^p < 0.001), and compared to RSV (^*^p < 0.05) and SRTAW04 (^*^p < 0.05) treated cultures.

To determine whether H_2_O_2_ treatment results in loss of mitochondrial membrane potential, and how this is affected by SIRT1 activator treatment, JC-1 staining was used. Staurosporine-differentiated RGC-5 cells were treated for 24 h with 500 μM H_2_O_2_ with or without 0.25 μM RSV or 5 μM SRTAW04, and live cell fluorescent microscopy pictures were taken after JC-1 staining. Results reveal minimal remaining red fluorescent labeling of viable, high membrane potential mitochondria in H_2_O_2_-treated cells as compared with healthy control cells (Figure 5). Furthermore, the ratio of green to red fluorescent JC-1 staining showed a significant increase in H_2_O_2_-treated cells compared with control cells. The green to red ratio was significantly lower in H_2_O_2_-treated cells that were also treated with RSV or SRTAW04, as compared to H_2_O_2_-treated cells without SIRT1 activators. Results indicate that H_2_O_2_ treatment disrupts the mitochondrial membrane potential, and this is prevented by treatment with RSV and SRTAW04. While JC-1 stain can be used to mark mitochondrial membrane potential, cell membrane potential may also be detected. Therefore, additional mitochondrial membrane potential detection was performed by staining with TMRM. Results further suggest that H_2_O_2_ treatment disrupts the mitochondrial membrane potential, and treatment with RSV and SRTAW04 prevents this (Figure 5).

### RSV and SRTAW04 require SIRT1 activity to prevent RGC-5 cell loss

**Figure 6:**
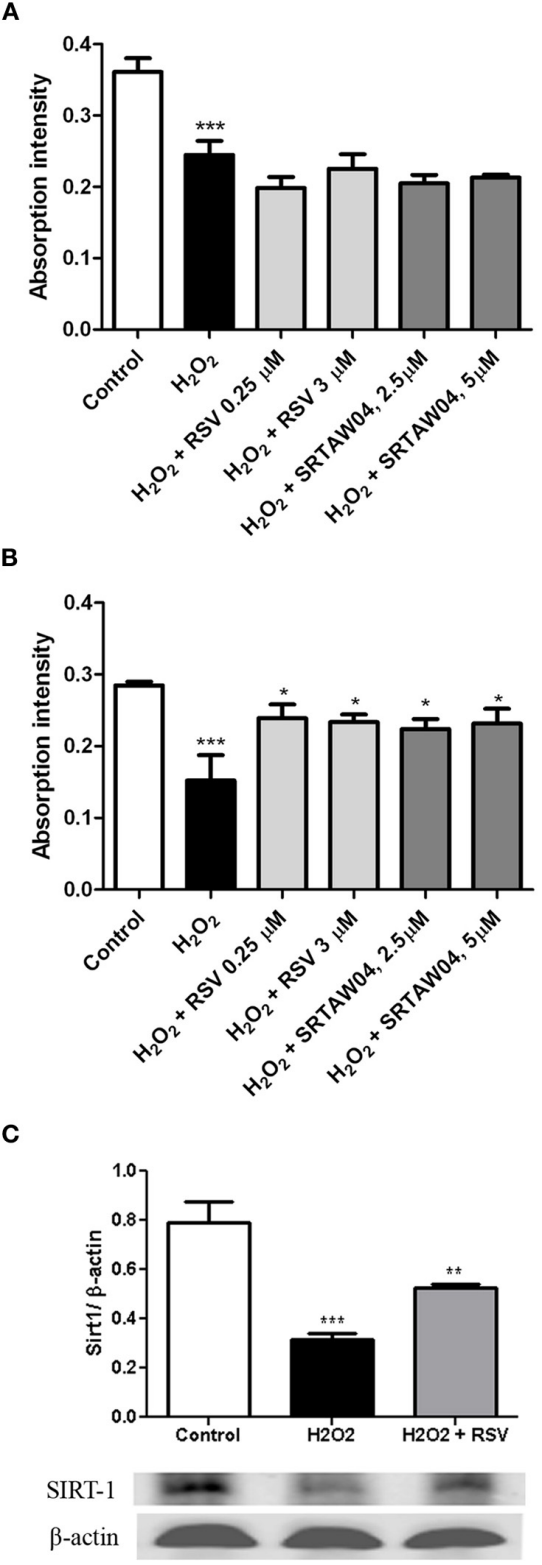
**RSV and SRTAW04 protection of RGC-5 cells is SIRT1-dependent.** Staurosporine-differentiated RGC-5 cells were transfected with SIRT1 siRNA **(A)** or a control siRNA **(B)** and cultured with or without 500 μM H_2_O_2_, and with or without RSV (0.25 and 3 μM) or SRTAW04 (2.5 and 5 μM) as indicated. SIRT1 siRNA blocked the ability of RSV and SRTAW04 to prevent loss of RGC-5 cells (^***^p < 0.001 vs. controls). This effect is specific to SIRT1, as RSV and SRTAW04 do prevent H_2_O_2_-induced RGC-5 loss in cells transfected with control siRNA (^*^p < 0.05). **(C)** Expression of SIRT1 was measured by Western blot of protein extracts from RSV-treated RGC-5 cultures not transfected with siRNA. Twenty-four hours after initiation of treatment, H_2_O_2_ shows a significant decrease of SIRT1 protein expression (^***^p < 0.001), whereas treatment with RSV significantly attenuates this decrease (^**^p < 0.01).

To confirm whether the ability of RSV and SRTAW04 to prevent loss of RGC-5 cells is dependent on SIRT1 activity, RGC-5 cells were transfected with SIRT1 siRNA or a control siRNA 24 h prior to addition of 500 μM H_2_O_2_ with or without RSV or SRTAW04. RGC-5 cell numbers were quantified 24 h later. SIRT1 siRNA blocked the ability of RSV and SRTAW04 to prevent RGC-5 cell loss (Figure 6A), whereas control siRNA did not (Figure 6B). SIRT1 protein expression was also measured by Western blotting of extracts from cultures treated with RSV. Results show H_2_O_2_ treatment leads to decreased SIRT1 expression, while RSV treatment significantly limits this decrease (Figure 6C).

### Effects of SIRT1 activators on expression of markers of mitochondrial and anti-oxidant function

**Figure 7:**
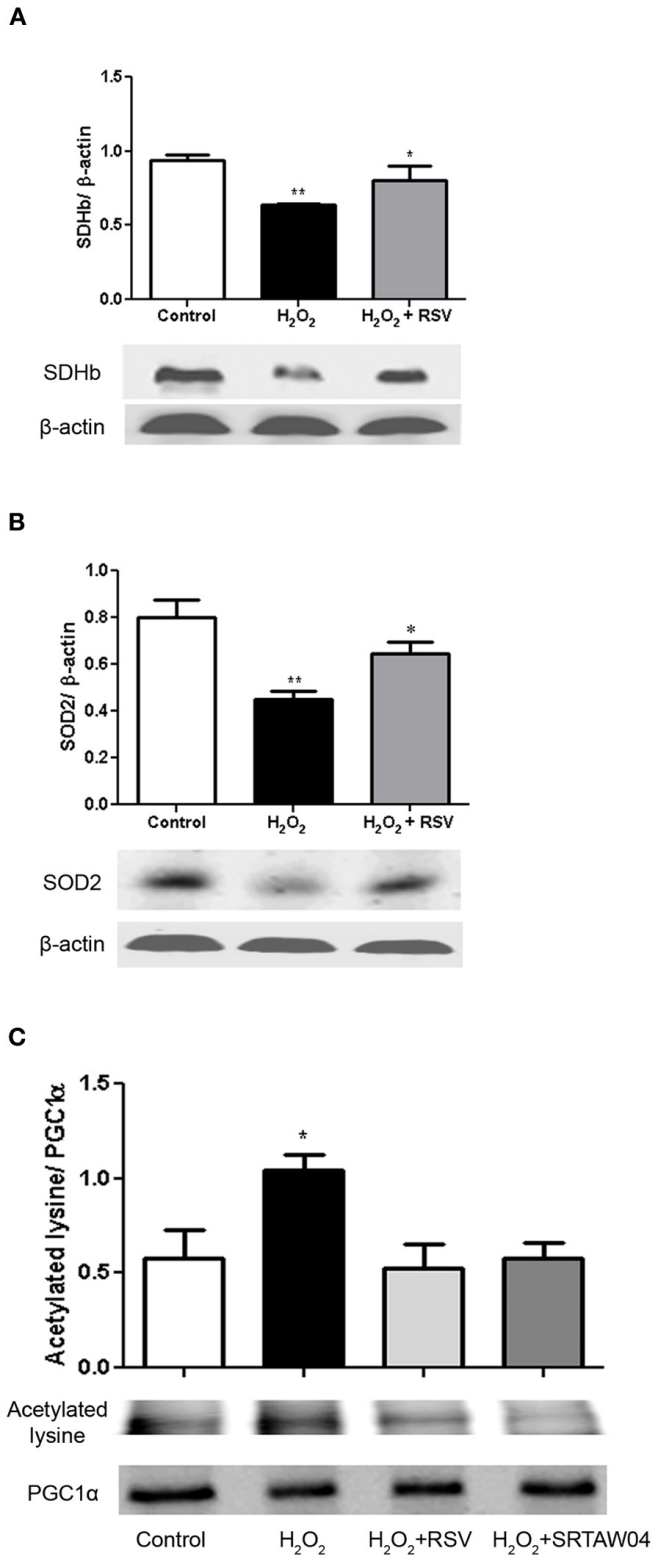
**RSV treatment increases markers of mitochondrial function in stressed RGC-5 cells.** Staurosporine-differentiated RGC-5 cells were cultured with or without 500 μM H_2_O_2_, and with or without 0.25 μM RSV for 24 h. **(A)** Western blot analysis shows a significant decrease in SDH expression (^**^p < 0.01) during H_2_O_2_ treatment which is attenuated by treatment with RSV (^*^p < 0.05). **(B)** Western blot analysis shows similar effects on SOD2 expression. The significant decrease (^**^p < 0.01) during H_2_O_2_ treatment, compared to controls, is not found in cells treated with RSV (^*^p < 0.05). **(C)** Protein extracts were immunoprecipitated with anti-PGC-1α antibodies, blotted, and hybridized with anti-PGC-1α and anti-acetylated lysine antibodies to assess the acetylation state of PGC-1α. H_2_O_2_ treatment significantly increases the proportion of acetylated PGC-1α (^*^p < 0.05) compared to controls, and RSV and SRTAW04 treatment each prevent this acetylation (^*^p < 0.05).

RGC-5 cells differentiated with staurosporine were stressed with 500 μM H_2_O_2_ for 24 h, with or without addition of 0.25 μM RSV, then protein extracts were generated for Western blotting. SDH functions not only in mitochondrial energy generation, but also has a role in oxygen sensing (Baysal, 2006). Protein levels of SDH show a significant decrease during H_2_O_2_ treatment which is attenuated by RSV (Figure 7A). We also measured the protein expression levels of SOD2, a mitochondrial protein which binds to superoxide byproducts of oxidative phosphorylation and converts them to hydrogen peroxide and diatomic oxygen (Suski et al., 2012). Results show a significant decrease in levels of SOD2 during H_2_O_2_ treatment, and treatment with RSV significantly limits this decrease (Figure 7B).

The peroxisome proliferator activated receptor (PPAR) co-activator 1-α (PGC-1α) is a transcriptional co-activator identified as an upstream regulator of mitochondrial number and function (Russell et al., 2004) and is activated by SIRT1-mediated deacetylation (Lagouge et al., 2006). RGC-5 cell protein extracts were immunoprecipitated with PGC-1α antibody. Western blot of the precipitated protein was probed with either PGC-1α antibody, or antibody to acetylated lysine, and the ratio of acetylated to deacetylated PGC-1α was compared. Results show a significant increase in acetylated lysine antibody immunoreactivity following H_2_O_2_ treatment, compared to control cells, as well as compared to RSV and SRTAW04 treated RGC-5 cells (Figure 7C). Overall PGC-1α expression showed no significant change between culture conditions. When compared, the decreased ratio of acetylated lysine to PGC-1α immunoreactivity in RSV and SRTAW04 treated cells demonstrates SIRT1 activators prevent an increase in PGC-1α acetylation.

### Resveratrol prevents loss of primary RGCs

**Figure 8:**
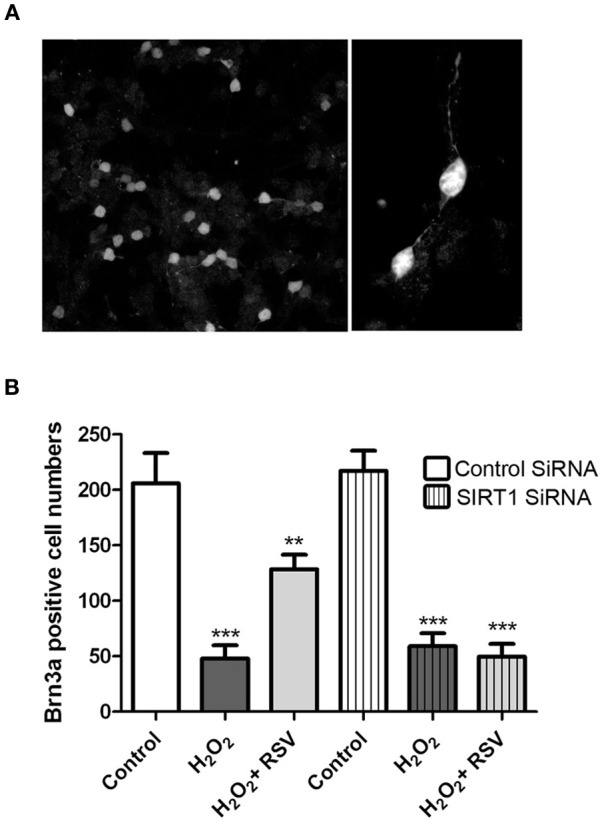
**SIRT1 activators prevent loss of primary RGCs.** Retinal cells were dissociated from neonatal mice and plated overnight prior to transfection with siRNA for 24 h and then treated 24 h with 500 μM H_2_O_2_ with or without 3 μM RSV. Cells were stained with antibodies to Brn3a to detect RGCs within mixed retinal cell cultures, and numbers of Brn3a positive cells were counted. **(A)** Brn3a staining demonstrates the presence of RGCs with neuronal morphology in these primary cultures. Original magnification × 20 (left) and × 63 (right). **(B)** H_2_O_2_ induces a significant decrease in the number of RGCs (^***^p < 0.001) which is attenuated by treatment with RSV (^**^p < 0.01) in the presence of control siRNA. SIRT1 siRNA transfection blocks the ability of RSV to attenuate RGC loss, as cell numbers remain significantly reduced compared to controls (^***^p < 0.001).

While RGC-5 cells differentiated with staurosporine exhibit some features of neurons, they are not a RGC specific cell line. Because optic neuritis and other optic neuropathies lead specifically to RGC loss, effects of RSV were further examined in primary retinal cell cultures, and RGC survival was assessed. Mixed dissociated retinal cells isolated from neonatal days 3 to 5 mice were incubated overnight, transfected for 24 h with SIRT1 specific or control siRNA, then treated for 24 h with 500 μM H_2_O_2_ with or without addition of RSV (3 μM). RGCs were identified within the mixed retinal cell cultures by immunohistochemical staining with antibodies to Brn3a (Figure 8A), and the number of RGCs was quantified by counting Brn3a positive cells by a blinded investigator. As seen in RGC-5 cells, H_2_O_2_ induced significant loss of primary RGCs, and RGC loss was reduced by treatment with RSV (Figure 8B). To determine whether the mechanism of this effect involves SIRT1 activity, primary RGC cultures were pretreated with SIRT1 siRNA. RSV neuroprotection of RGCs was blocked by SIRT1 siRNA but not by control siRNA (Figure 8B).

## Discussion

In the current studies, we demonstrated that two SIRT1 activating compounds, RSV, a naturally occurring plant polyphenol (Siemann and Creasey, 1992; Dong, 2003), and SRTAW04, a compound that activates SIRT1 at an order of magnitude lower concentration, similar to other SIRT1 activators (Milne et al., 2007), both significantly reduce loss of RGC-5 cells in response to several different stressors. SRTAW04 is a member of the same family and exhibits similar chemical properties of SIRT1 activation as the previously described compound 14 in studies by Dai and colleagues (Dai et al., 2010; Dr. Robert Perni, pers. communication). Results in the current study demonstrating that SIRT1 siRNA blocks the protective effects on stressed cells are also consistent with SRTAW04 and RSV functioning through SIRT1 activity in RGC-5 cells. While RSV is a known activator of SIRT1, treatment also resulted in preserved expression of SIRT1 which may explain or at least contribute to SIRT1-mediated neuroprotection.

A common feature of RGC-5 cell loss was increased oxidative stress, as marked by accumulation of superoxide. SIRT1 activating compounds significantly reduced the level of ROS within cells, suggesting that reduction of oxidative stress is one major mechanism of SIRT1 activator-mediated neuroprotection. Attenuation of RSV and SRTAW04 neuroprotective effects by a known SIRT1 inhibitor, NAM, further suggests that the mechanism of these effects is dependent on the ability of these compounds to activate SIRT1. Interestingly, NAM has been found to be neuroprotective in some neuronal injury models, due to its ability to inhibit PARP1 (Liu et al., 2009). Those studies used much higher concentrations of NAM than in the current the studies, which may explain why NAM alone had no effect on RGC-5 cells.

We previously showed that RSV and other SIRT1 activators reduce RGC loss in EAE models of optic neuritis (Shindler et al., 2007, 2010; Fonseca-Kelly et al., 2012), and therefore provide an important potential therapy to prevent the neuronal damage that leads to permanent neurologic disability in optic neuritis and MS patients. To further elucidate the mechanism by which SIRT1 activators are able to prevent RGC loss, in the present study we investigated effects of two SIRT1 activating compounds on differentiated and non-differentiated neuronal cultures. However, while original reports suggested the RGC-5 cell line is a transformed proliferating cell line that expresses some RGC-specific markers (Krishnamoorthy et al., 2001; Wood et al., 2010), it is important to note that the lineage and characteristics of this cell line has since been found to be different (Van Bergen et al., 2009), with features of photoreceptors, and limits the relevance of current results to optic nerve disease. For most of our experiments we used differentiated RGC-5 cells because sub-lethal treatment with staurosporine induces differentiation of RGC-5 cells to a non-mitotic phenotype with neurite outgrowth and expression of at least one neuronal ion channel (Frassetto et al., 2006), making them a better model for the study of neuronal cell properties than non-differentiated RGC-5 cells. Importantly, SIRT1-dependent protective effects of RSV were also found in primary retinal cultures, suggesting that the current findings may be indicative of RGC responses and have the potential to translate to mechanisms active in optic nerve diseases.

Oxidative stress plays an important role in the pathophysiology of optic neuritis. Studies have demonstrated that inflammatory cells act as mediators of optic nerve injury by release of ROS into the extracellular microenvironment (Guy et al., 1994), with accumulation of ROS in RGC axons in the optic nerve (Qi et al., 2007). Consistent with these previous studies, we found an increase in the superoxide ROS in optic nerve sections of EAE mice. Furthermore, we showed that different stressors, serum starvation, doxorubicin, and H_2_O_2_, induce a similar significant increase in ROS levels in RGC-5 cell cultures, making this a useful model for studying mechanisms of neuronal loss and neuroprotection. The specific use of H_2_O_2_ in RGC-5 cell and primary RGC cultures may be especially relevant because prior reports showed that H_2_O_2_ plays a major role in the pathogenesis of optic neuritis (Guy et al., 1993), marked by the presence of H_2_O_2_ in optic nerves of animals with acute EAE, and during the primary stage of demyelination, the optic nerve head is a site of H_2_O_2_ localization derived from sensitized inflammatory cells.

Mitochondria are the primary source of cellular ROS. Increased ROS activity is linked to neurodegenerative diseases that have axonal and neuronal loss as a major feature (Kalman and Leist, 2003). In addition, SIRT1 activation has been shown previously to promote mitochondrial biogenesis in some tissues, including muscle (Lagouge et al., 2006). Thus, we hypothesized that the mechanism of SIRT1 activator-mediated reduction of oxidative stress in RGC-5 cells occurs due to promotion of mitochondrial function, as well as due to increased levels of enzymes that reduce oxidative stress. Consistent with this, we found that SIRT1 activators induce a significant increase in SOD2 in RGC-5 cells. SOD2 is a crucial scavenger for superoxide in mitochondria. SOD2 converts superoxide to H_2_O_2_ and mitochondrial glutathione peroxidase GPx1 catalyzes the reduction of H_2_O_2_ to H_2_O (Li et al., 2000). Increase in SOD2 expression is a downstream target of SIRT1, and consistent with our results, previous studies showed SIRT1-induced superoxide dismutase, which was further enhanced by RSV, increased the resistance of C2C12 myoblasts to oxidative stress (Tanno et al., 2010).

We also found SIRT1 activators increase SDH expression in stressed RGC-5 cells. SDH not only plays a central role in the Krebs cycle and the respiratory chain, but it also differs from other mitochondrial dehydrogenases due to its unique redox properties (Balietti et al., 2009). In partnership with ubiquinone, SDH represents a crucial antioxidant enzyme in mitochondria controlling superoxide scavenging activity of the respiratory chain. When succinate-ubiquinone activity is inhibited, electrons that would normally transfer through the SDHB subunit to the ubiquinone pool are instead transferred to O_2_ to create ROS such as superoxide (Rustin et al., 2002). It has been suggested that the SDHB mutation results in a complete loss of electron transport chain complex II activity in mitochondria (Gimenez-Roqueplo et al., 2002). As a possible protective mechanism, we investigated the preservation of mitochondrial membrane potential using JC-1 and TMRM staining. Oxidative stress alters mitochondrial permeability by opening transition pores, and collapses the mitochondrial membrane potential (Niimi et al., 2012), a major driving force for oxidative phosphorylation. Under chronic oxidative stress, mitochondria can be subject to irreversible increase in permeability of the inner mitochondrial membrane resulting in collapse of membrane potential, and the current data showing preserved JC-1 and TMRM staining suggest that SIRT1 activators prevent this collapse in stressed RGC-5 cells.

Finally, SIRT1 activation induces mitochondrial biogenesis in various tissues (Lagouge et al., 2006; Rasbach and Schnellmann, 2008; Gurd et al., 2009). Mitochondrial biogenesis is a highly regulated process operating through PGC-1α-dependent nuclear respiratory factors. PGC-1α is heavily acetylated by acetyltransferase GCN5 whereas it is deacetylated by SIRT1 (Gerhart-Hines et al., 2007). SIRT1-mediated deacetylation and activation of PGC-1α therefore serves as an important response of the cell to increase mitochondrial metabolism.

Together, results demonstrate that SIRT1 activation prevents accumulation of ROS and cell loss, suggesting that this is one mechanism that SIRT1 activators can use to prevent neuronal loss. Results indicate that the mechanism of RSV effects involve SIRT1 activity, and associated deacetylation of PGC-1α is likely to be involved in the positive regulation of oxidative metabolism, and co-regulates the induction of other proteins like SDH and SOD2 that participate in the cellular response to oxidative stress. The common mechanism of oxidative stress found in many neurodegenerative processes suggests that SIRT1 activators may exert similar anti-oxidative effects as potential neuroprotectants in a variety of neurodegenerative diseases.

### Conflict of interest statement

The authors declare that the research was conducted in the absence of any commercial or financial relationships that could be construed as a potential conflict of interest.
